# Sustained high glucose exposure sensitizes macrophage responses to cytokine stimuli but reduces their phagocytic activity

**DOI:** 10.1186/s12865-018-0261-0

**Published:** 2018-07-11

**Authors:** Sofia Pavlou, Jaime Lindsay, Rebecca Ingram, Heping Xu, Mei Chen

**Affiliations:** Centre for Experimental Medicine, School of Medicine, Dentistry & Biomedical Science, Queen’s University Belfast, Belfast, UK

**Keywords:** Macrophages, Diabetes, Cytokine, Phagocytosis, Glycolysis

## Abstract

**Background:**

Macrophages are tissue resident immune cells important for host defence and homeostasis. During diabetes, macrophages and other innate immune cells are known to have a pro-inflammatory phenotype, which is believed to contribute to the pathogenesis of various diabetic complications. However, diabetic patients are highly susceptible to bacterial infections, and often have impaired wound healing. The molecular mechanism underlying the paradox of macrophage function in diabetes is not fully understood. Recent evidence suggests that macrophage functions are governed by metabolic reprograming. Diabetes is a disorder that affects glucose metabolism; dysregulated macrophage function in diabetes may be related to alterations in their metabolic pathways. In this study, we seek to understand the effect of high glucose exposure on macrophage phenotype and functions.

**Results:**

Bone marrow cells were cultured in short or long term high glucose and normal glucose medium; the number and phenotype of bone marrow derived macrophages were not affected by long-term high glucose treatment. Short-term high glucose increased the expression of IL-1β. Long-term high glucose increased the expression of IL-1β and TNFα but reduced the expression of IL-12p40 and nitric oxide production in M1 macrophage. The treatment also increased Arg-1 and IL-10 expression in M2 macrophages. Phagocytosis and bactericidal activity was reduced in long-term high glucose treated macrophages and peritoneal macrophages from diabetic mice. Long-term high glucose treatment reduced macrophage glycolytic capacity and glycolytic reserve without affecting mitochondrial ATP production and oxidative respiration.

**Conclusion:**

Long-term high glucose sensitizes macrophages to cytokine stimulation and reduces phagocytosis and nitric oxide production, which may be related to impaired glycolytic capacity.

**Electronic supplementary material:**

The online version of this article (10.1186/s12865-018-0261-0) contains supplementary material, which is available to authorized users.

## Background

Macrophages are tissue resident immune cells important for host defence and homeostasis. During inflammation, circulating monocytes are recruited and differentiated into macrophages at the site of tissue damage and infection. The key functions of these macrophages are to eliminate insults or pathogens and to promote tissue repair. During diabetes, macrophages and other innate immune cells are known to have a pro-inflammatory phenotype, which is believed to contribute to the pathogenesis of various diabetic complications, including diabetic nephropathy, atherosclerosis, and retinopathy [1]. Uncontrolled monocyte and neutrophil activation may contribute to diabetes-mediated vascular damage through abnormal leukocyte-endothelial interaction or leukostasis [2, 3]. Inhibition of inflammation has been proposed as a therapeutic approach for diabetic complications [4, 5]. However, diabetic patients are highly susceptible to bacterial infections, and often have impaired wound healing [6]. It is believed that this is partially related to the reduced bactericidal function and wound healing capacity of innate immune cells in diabetic patients. The molecular mechanism underlying the paradox of macrophage function in diabetes is not fully understood.

Recent evidence suggests that macrophage functions are governed by metabolic reprograming [7]. Metabolic pathways not only provide energy in the form of adenosine triphosphate (ATP), but also regulate macrophage functions [7]. For example, during acute inflammation, the classically activated macrophages (M1) predominately utilize glycolysis, but not mitochondrial oxidative phosphorylation, to support their inflammatory function [8]. This metabolic pathway generates amino acids, ribose and nicotinamide adenine dinucleotide phosphate (NADPH) that are necessary for cytokine synthesis, DNA replication and production of reactive oxygen species (ROS) [7]. In addition, glycolytic enzymes and intermediate products are actively involved in the regulation of cytokine production. The pyruvate kinase M2 (PKM2) has been shown to induce IL-1β expression through activating hypoxia inducible factor 1, alpha subunit (HIF1α) [8], whereas succinate enhances the stabilization of HIF1α and supports IL-1β expression [9]. Since diabetes is a disorder that affects glucose metabolism, we seek to understand whether dysregulated macrophage function in diabetes is related to alterations in their metabolic pathways.

In this study, we investigated the effect of high glucose on bone marrow cell proliferation and differentiation into macrophages. We further investigated the metabolic pathways of macrophages generated under high glucose and normal glucose conditions and how this is related to their functions. We found that sustained high glucose treatment impairs the glycolysis pathway in macrophages, which may be related to their reduced bactericidal activity and ROS production.

## Results

### The effect of long-term high glucose on bone marrow cell proliferation and differentiation

**Fig. 1:**
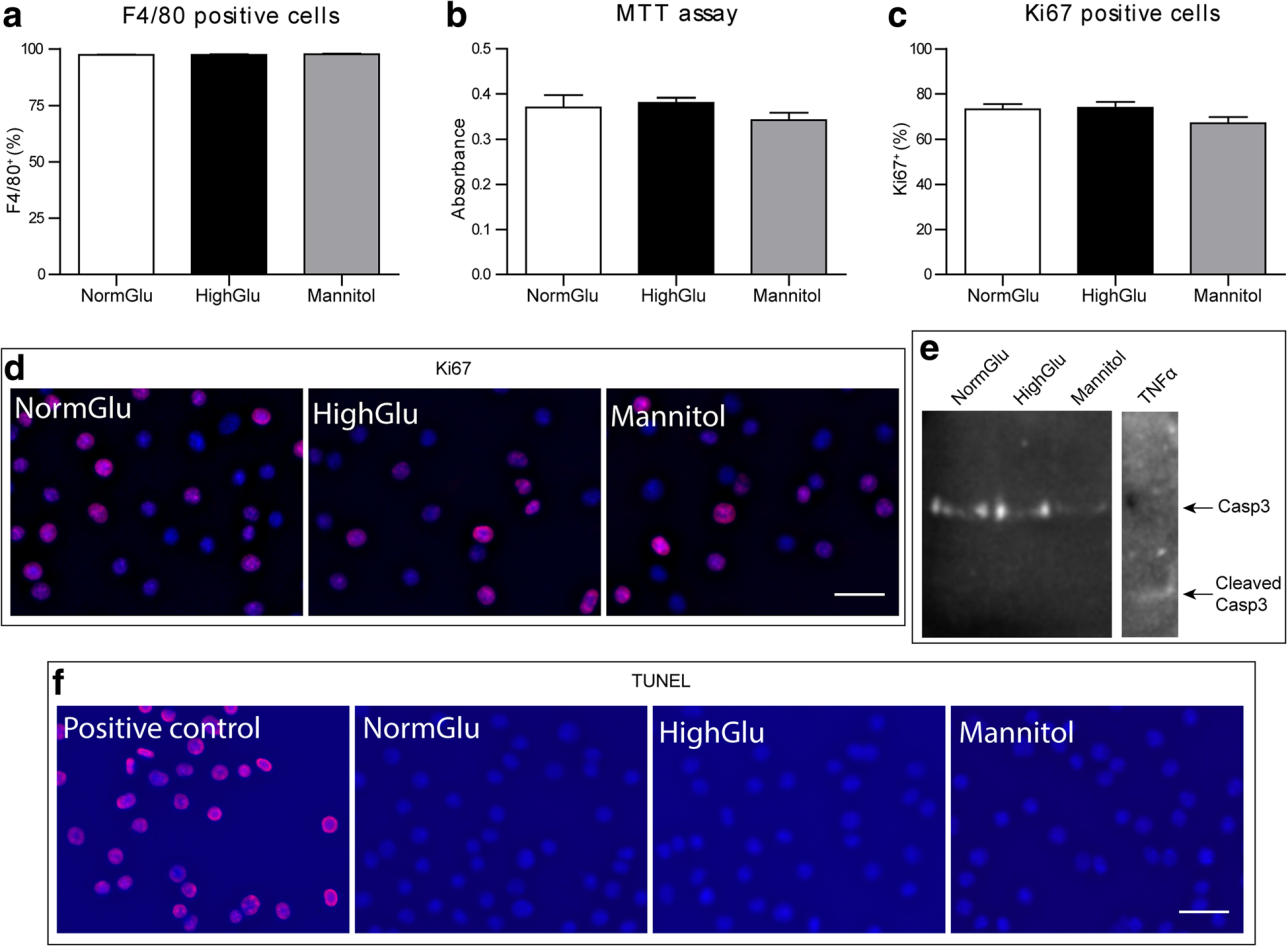
Long-term HighGlu does not affect differentiation, viability or proliferation of BMDMs. Bone marrow cells were cultured for 7 days under NormGlu, HighGlu or Mannitol (osmolarity control) conditions. **a** Flow cytometric analysis showing the percentage of F4/80^+^ cells differentiated under the three culture conditions. **b** MTT assay demonstrating the cell viability of BMDMs. **c** Graph showing the percentage of Ki67^+^ cells. **d** Representative images of Ki67^+^ cells (red), counterstained with DAPI (blue). **e** Western blot for Casp3 using protein extracts from the three culture conditions. BMDMs treated with TNFα were used as a positive control. **f** Representative images of TUNEL assay (red). DNase-treated BMDMs were used as a positive control. Scale bars = 25 μm. Data are represented as mean ± SEM. One-way ANOVA followed by Tukey’s multiple comparison post hoc test was performed

In diabetes, myeloid progenitor cells are subjected to sustained high glucose conditions. To investigate the impact of the sustained hyperglycaemic environment on macrophage progenitors, we generated BMDMs under high glucose DMEM (HighGlu) conditions. Flow cytometric analysis showed that over 96% of cells were F4/80^+^ (Fig. 1a). The percentage and absolute number of F4/80^+^ macrophages were similar in BMDMs generated under normal glucose DMEM ( NormGlu) and HighGlu conditions (Fig. 1a). MTT assay further confirmed that the cell numbers from different groups were also similar (Fig. 1b).

Ki67 is a nuclear protein necessary for cell proliferation [10]. There was no significant difference in the percentage of Ki67^+^ cells over the total number of cells (DAPI^+^) between the different treatment groups (Fig. 1c, d). Western blot did not detect the cleaved form of Caspase-3 (Fig. 1e) in any of the experimental groups. No apoptotic cells were detected in the treatment groups by TUNEL staining (Fig. 1f). Our results suggest that long-term HighGlu treatment did not affect macrophage differentiation from bone marrow cells and did not induce macrophage apoptosis.

### Short-term and long-term high glucose treatment on BMDM immune gene expression

**Fig. 2:**
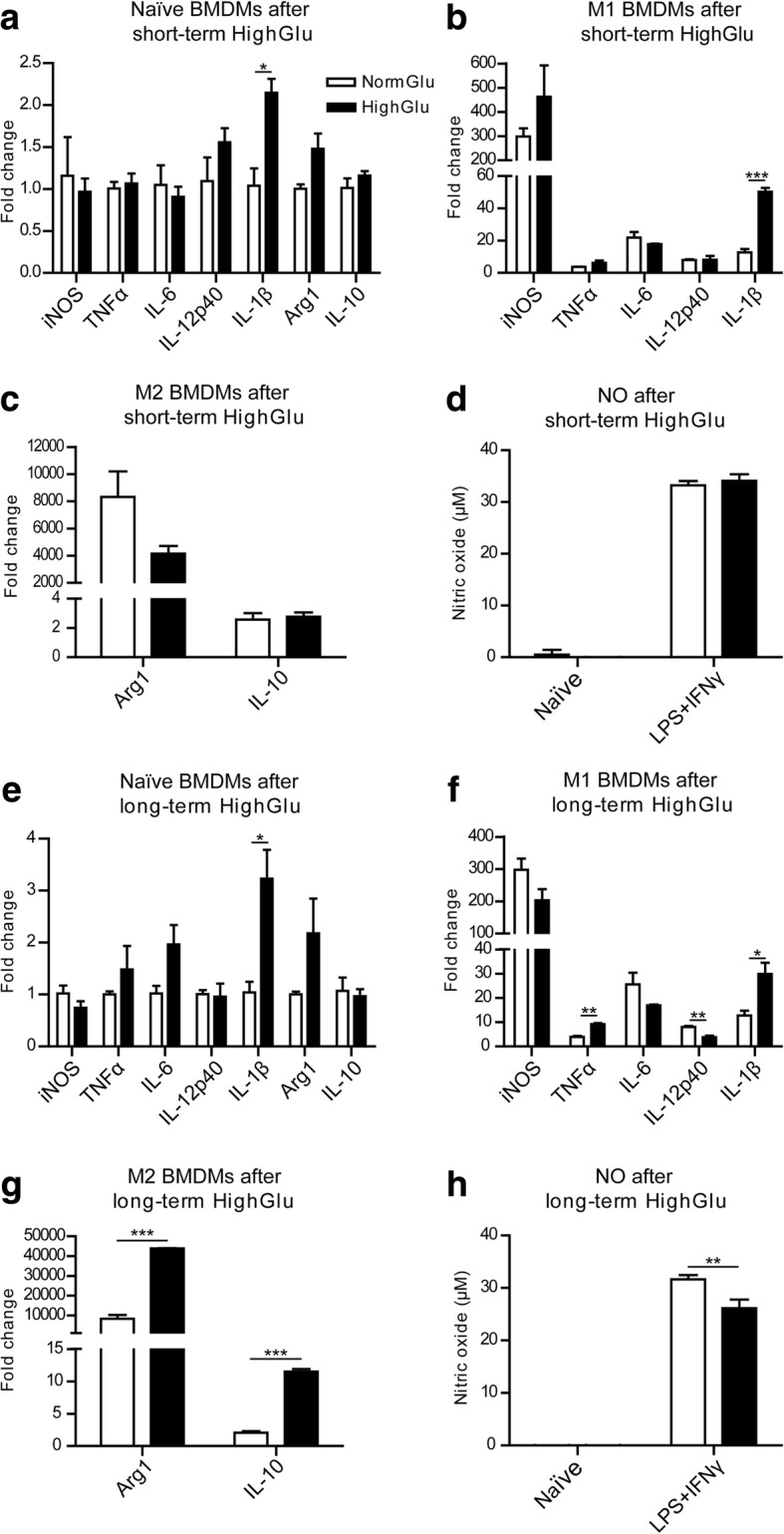
The effects of short and long-term HighGlu on BMDM gene expression and NO production. Bone marrow cells were cultured for 7 days under NormGlu (white bars) or HighGlu (black bars) conditions. They were then plated and stimulated with LPS + IFNγ (M1) or IL-4 (M2) for 24 h. For short-term HighGlu, cells differentiated under NormGlu, were exposed to HighGlu for 24 h along with M1 or M2 stimulation. **a-c** qRT-PCR showing the expression of immune-related genes in naïve (**a**), LPS + IFNγ (**b**) or IL-4 (**c**) treated BMDMs after short-term exposure to HighGlu treatment. **d** Graph showing the concentration of NO in supernatants of naïve and LPS + IFNγ-treated BMDMs under NormGlu or short-term HighGlu conditions. **e-g** qRT-PCR showing the expression of immune-related genes in naïve (**e**), LPS + IFNγ (**f**) or IL-4 (**g**) treated BMDMs after long-term exposure to HighGlu treatment. **h** NO concentration in the supernatants of naïve and LPS + IFNγ stimulated BMDMs after long-term HighGlu. Data are represented as mean ± SEM. Unpaired, two-tailed Student’s t test was performed. * *p* < 0.05, ** *p* < 0.01, *** *p* < 0.001

Next, we investigated the impact of HighGlu on macrophage gene expression. The exposure of naïve BMDMs generated under NormGlu to HighGlu for 24 h (short-term HighGlu) increased IL-1β expression but did not affect the expression of inducible nitric oxide synthase 2 (iNOS), tumour necrosis factor-α (TNFα), interleukin 6 (IL-6), interleukin 12p40 (IL-12p40), arginase-1 (Arg-1), and interleukin 10 (IL-10) (Fig. 2a). When M1 macrophages were exposed to HighGlu for 24 h, the expression of IL-1β was significantly increased (Fig. 2b). Short-term HighGlu treatment did not affect other genes expression in both M1 (Fig. 2b) and M2 macrophages (Fig. 2c). NO production was also not affected (Fig. 2d). The results suggest that short-term HighGlu enhances macrophage pro-inflammatory response.

BMDMs generated under long-term HighGlu condition had significantly higher levels of IL-1β expression, but the expression of other immune genes such as iNOS, TNFα, IL-6, IL-12p40, Arg-1 and IL-10 was not affected compared with BMDMs generated under NormGlu conditions (Fig. 2e). Following M1 polarization, BMDMs generated under long-term HighGlu conditions expressed lower levels of IL-12p40, but higher levels of TNFα and IL-1β compared to M1 BMDMs under NormGlu conditions (Fig. 2f). Long-term HighGlu treatment also reduced the expression of iNOS, although not significantly (Fig. 2f). In contrast with the iNOS expression, NO production was significantly lower in long-term HighGlu treated M1 macrophages (Fig. 2h). Interestingly, IL4-induced upregulation of Arg-1 and IL-10 was significantly enhanced in long-term HighGlu treated BMDMs (Fig. 2g). Mannitol did not affect the expression levels of the aforementioned cytokines in BMDMs (data not shown). The results suggest that long-term HighGlu may alter macrophage response to cytokine stimulations. In response to LPS + IFNγ stimulation, they have reduced ability to generate NO, but have increased TNFα and IL-1β expression. They also appear to be more sensitive to Th2-type cytokine (e.g., IL-4) stimulation.

**Fig. 3:**
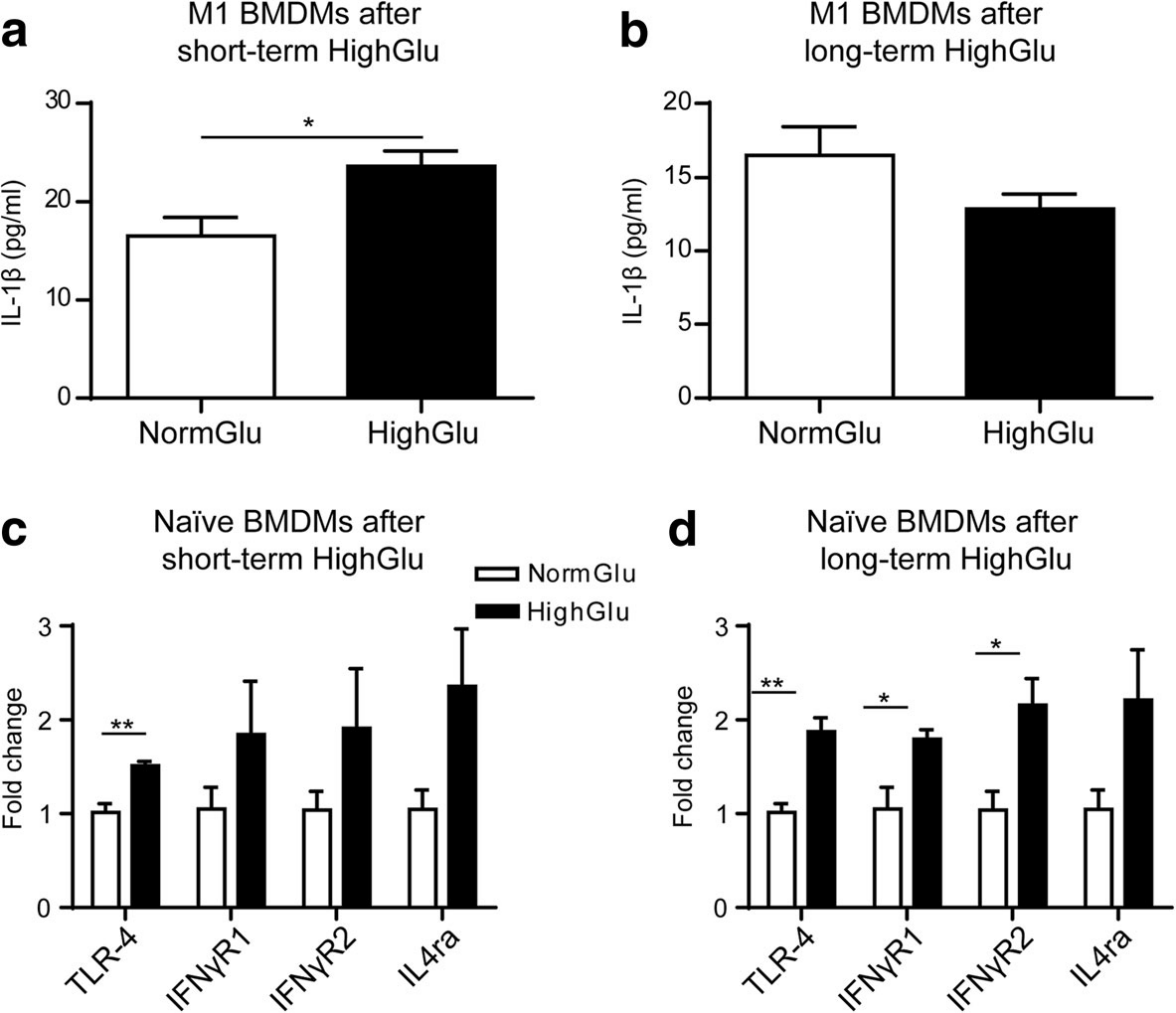
The effects of short- and long-term HighGlu on IL-1β secretion and LPS/cytokine receptor expression. Bone marrow cells were cultured for 7 days under NormGlu (white bars) or HighGlu (black bars) conditions. They were then plated and stimulated with LPS + IFNγ (M1) for 24 h. For short-term HighGlu, cells differentiated under NormGlu, were exposed to HighGlu for 24 h along with M1 stimulation. **a-b** ELISA showing the levels of IL-1β in the supernatants of M1 BMDMs exposed to short-term (**a**) or long-term (**b**) HighGlu. **c-d** qRT-PCR showing the expression of LPS and cytokine receptors in naïve BMDMs exposed to short-term (**c**) or long-term (**d**) HighGlu. Data are represented as mean ± SEM. Unpaired, two-tailed Student’s t test was performed. * *p* < 0.05, ** *p* < 0.01

To investigate if the increased IL-1β mRNA expression in HighGlu-treated macrophages resulted in higher levels of protein secretion, IL-1β levels in culture supernatants were measured using ELISA. The results show that the levels of IL-1β are undetectable in naïve BMDMs (data not shown). LPS + IFNγ stimulation and high glucose significantly increased IL-1β production (Fig. 3a-b).

Since macrophages generated under NormGlu and HighGlu conditions responded differently to LPS + IFNγ and IL-4 stimulations, we investigated whether they expressed different levels of relevant receptors. Short-term HighGlu only increased Toll-like receptor 4 (TLR-4) expression in BMDMs (Fig. 3c), whereas long-term HighGlu significantly increased the expression of TLR-4, interferon gamma receptor 1 (IFNγR1) and interferon gamma receptor 2 (IFNγR2) (Fig. 3d). Interestingly, the expression levels of interleukin 4 receptor, alpha (IL4ra) remained unchanged in both short-term and long-term HighGlu treated macrophages (Fig. 3c-d).

### The effect of long-term high glucose on BMDM phagocytosis and bactericidal function

**Fig. 4:**
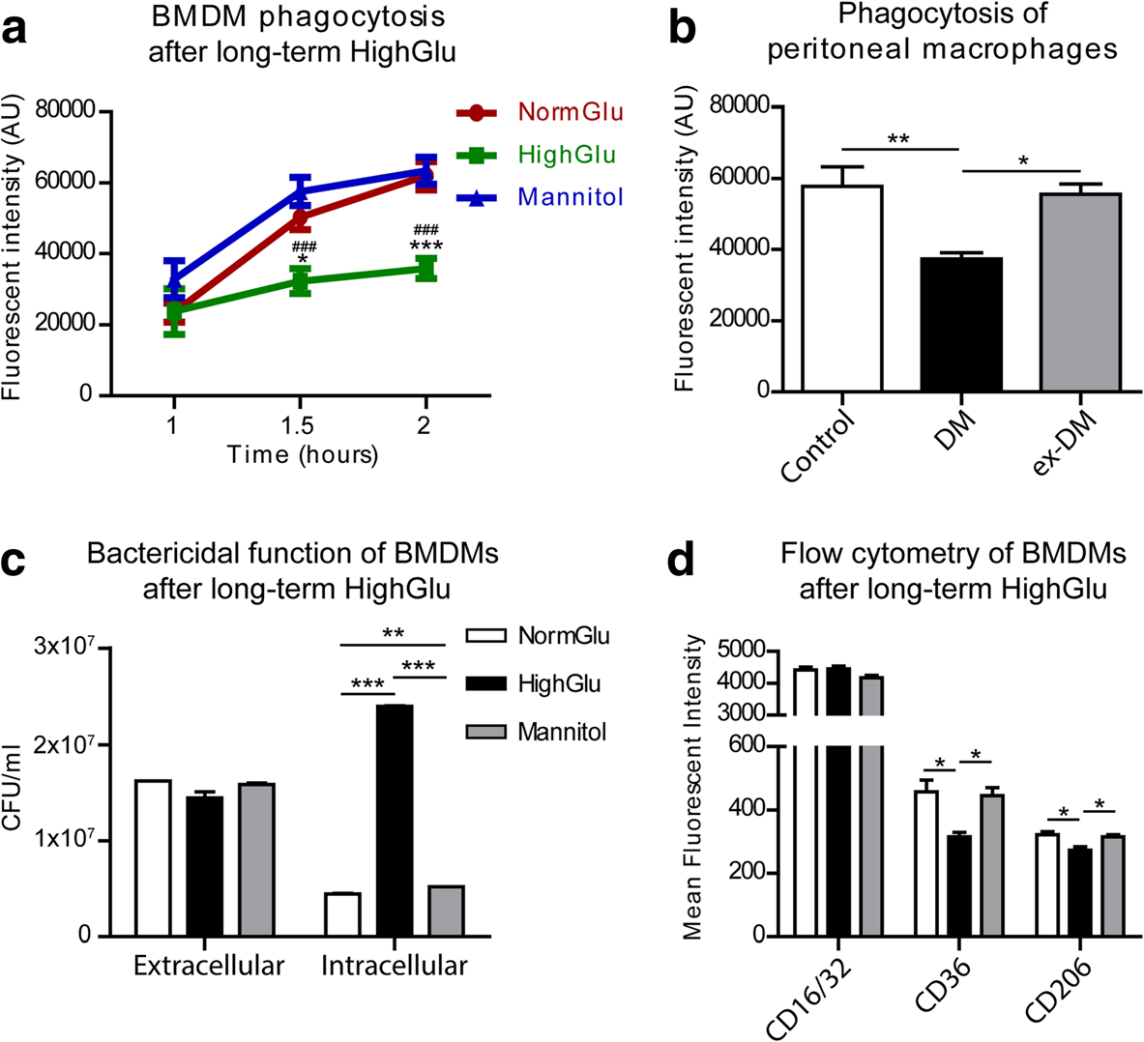
Long-term HighGlu and diabetes mellitus affect the phagocytic and bactericidal functions of macrophages. Bone marrow cells were cultured for 7 days under NormGlu, HighGlu or Mannitol conditions. Peritoneal macrophages were isolated from control, diabetic (DM) or ex-diabetic (ex-DM) mice. Phagocytosis was assessed using pHrodo *S. aureus* bioparticles and bactericidal function using *P. aeruginosa* PAO1 cultures. The expression of CD16/32, CD36 and CD206 was measured by flow cytometry. **a** Phagocytic activity of BMDMs cultured under NormGlu (red), HighGlu (green) or Mannitol (blue) conditions. **b** Phagocytic activity of peritoneal macrophages. **c** Bactericidal function of BMDMs. **d** Mean fluorescence intensity of CD16/32, CD36 and CD206 in BMDMs. White bars: NormGlu, black bars: HighGlu, grey bars: Mannitol. Data are represented as mean ± SEM. Two-way ANOVA with bonferroni correction was performed in (**a**); * *p* < 0.05, *** *p* < 0.001 relative to NormGlu, ^♯♯♯^ *p* < 0.001 relative to Mannitol. One-way ANOVA followed by Tukey’s multiple comparison post hoc test was performed in (**b-d**); * *p* < 0.05, ** *p* < 0.01, *** *p* < 0.001

The evident difference between short-term and long-term HighGlu on macrophage response to cytokine stimulation prompted us to further investigate other macrophage functions such as phagocytosis. Using the pHrodo *S. aureus* bioparticles phagocytosis assay, we did not observe any difference between macrophages treated with or without short-term HighGlu (Additional file 1: Figure S1). However, in macrophages generated under HighGlu significantly reduced phagocytosis was observed after 1.5 h and 2 h of bioparticle incubation compared with BMDMs in NormGlu and Mannitol conditions (Fig. 4a). Long-term Mannitol treatment did not affect macrophage phagocytosis (Fig. 4a).

To further confirm the effect of long-term HighGlu on macrophage phagocytic function, we harvested elicited peritoneal macrophages from STZ-induced diabetic mice, ex-diabetic mice and healthy controls and performed the same phagocytosis assay. Peritoneal macrophages from diabetic mice showed significantly reduced phagocytic capacity when compared to age-matched healthy control mice (Fig. 4b). Interestingly, peritoneal macrophages from ex-diabetic mice had normal levels of phagocytosis, suggesting that hyperglycaemia-induced phagocytosis impairment is reversible.

The pHrodo *S. aureus* bioparticles assay not only detects the uptake of bacterial particles, but also measures the formation of phagolysosomes, a pivotal action by macrophage to kill bacteria. The bactericidal function of macrophages was further assessed by challenging macrophages with live pathogenic bacteria. After 1 h incubation of *P. aeruginosa* PAO1 with BMDMs, extracellular as well as intracellular bacteria were quantified. Extracellular bacterial colonies were comparable between the different groups (Fig. 4c), suggesting that the same number of bacteria were taken up by the different macrophages. Interestingly, the colonies of intracellular surviving bacteria in macrophages generated under long-term HighGlu conditions were significantly higher than those in macrophages generated under NormGlu and Mannitol conditions (Fig. 4c). The results suggest that long-term HighGlu impairs bactericidal ability of macrophages.

To understand if the reduced phagocytosis in BMDMs generated under HighGlu conditions seen at 1.5 h and 2 h (Fig. 4a) is related to altered scavenger receptor expression, the expression of cell surface receptors CD16/32, CD36 and CD206 in different types of BMDMs was examined by flow cytometry. Our results show that the expression levels of CD16/CD32 were comparable (Fig. 4d). However, the expression of CD36 and CD206 was significantly lower in BMDMs cultured under HighGlu conditions compared to those cultured under NormGlu or Mannitol conditions (Fig. 4d).

### The effect of long-term high glucose on macrophage glucose metabolism and mitochondrial oxygen consumption

**Fig. 5:**
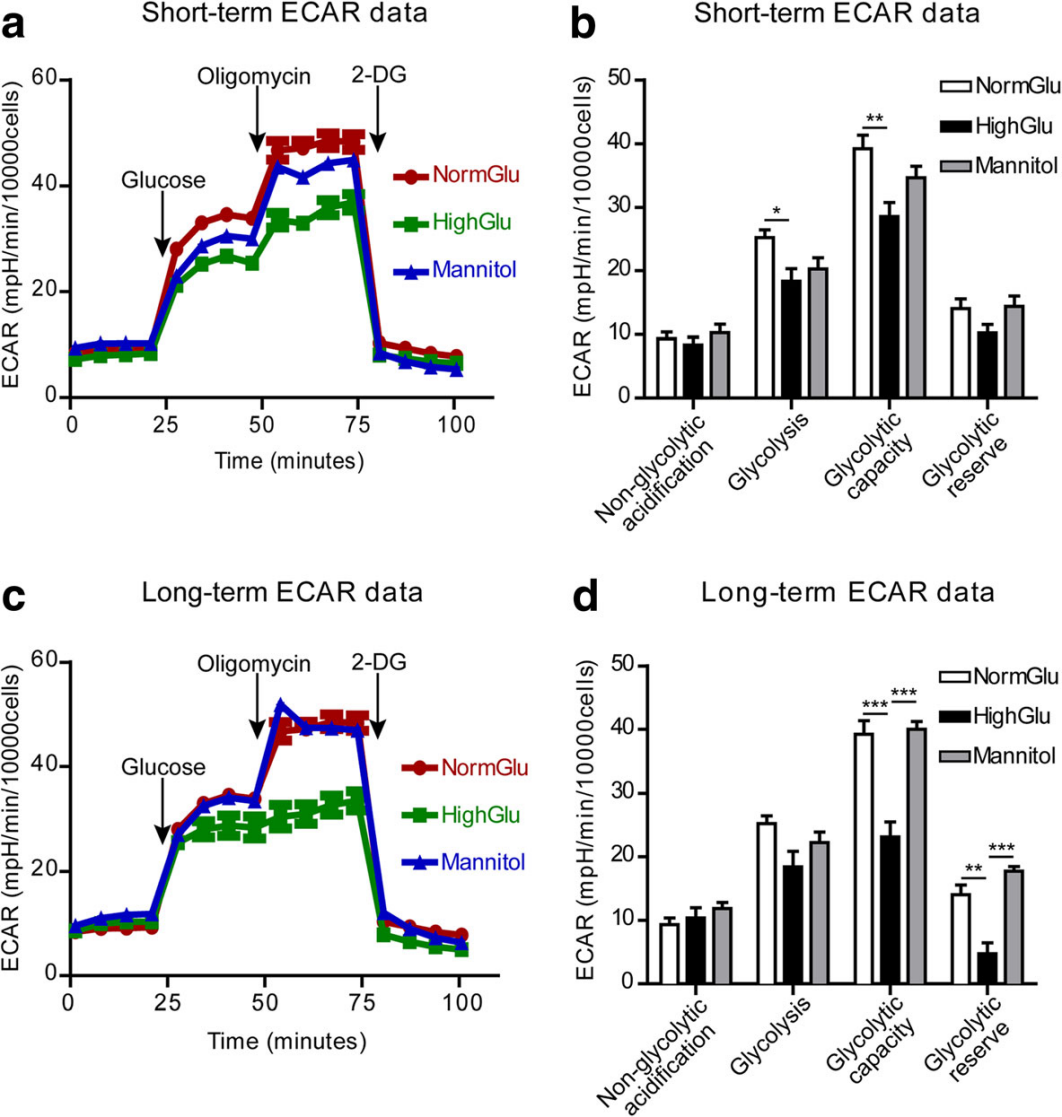
HighGlu treatments affect the glycolytic pathway of BMDMs. Bone marrow cells were cultured for 7 days under NormGlu, HighGlu or Mannitol conditions (long-term treatments). For short-term HighGlu and Mannitol, cells differentiated under NormGlu, were exposed to HighGlu or Mannitol for 24 h, respectively. The glycolysis stress assay was performed using the Seahorse XFe96 analyzer. **a** Representative profile after glycolysis stress assay showing the ECAR of BMDMs exposed to short-term NormGlu (red), HighGlu (green) or Mannitol (blue). **b** Graph showing non-glycolytic acidification, glycolysis, glycolytic capacity and glycolytic reserve of BMDMs after short-term NormGlu (white bars), HighGlu (black bars) and Mannitol (grey bars) treatments. **c** Representative profile after glycolysis stress assay showing the ECAR of long-term treated BMDMs. **d** Graph showing non-glycolytic acidification, glycolysis, glycolytic capacity and glycolytic reserve of BMDMs after long-term exposure to the different conditions. Data are represented as mean ± SEM. One-way ANOVA followed by Tukey’s multiple comparison post hoc test was performed; * *p* < 0.05, ** *p* < 0.01, *** *p* < 0.001

Immune cells’ function is governed by their metabolisms [11]. We hypothesize that long-term HighGlu treatment may affect macrophage function through regulating their metabolic pathways. In the Glycolysis Stress assay, BMDMs were incubated in basal medium (medium without added glucose, supplemented with glutamine) for 1 h followed by the various stimulations depicted in Fig. 5a. The non-glycolytic (baseline) extracellular acidification rate (ECAR) of naïve BMDMs in all culture conditions was similar (Fig. 5a). The addition of glucose and subsequently oligomycin resulted in a rapid increase in ECAR (Fig. 5a). Both short-term (24 h, Fig. 5a, b) and long-term (7 days, Fig. 5c, d) HighGlu treatment significantly reduced BMDM response to glucose and oligomycin stimulations. Specifically, the short-term HighGlu treatment reduced glycolysis and glycolytic capacity (Fig. 5b); whereas long-term HighGlu reduced glycolytic capacity and glycolytic reserve (Fig. 5d).

**Fig. 6:**
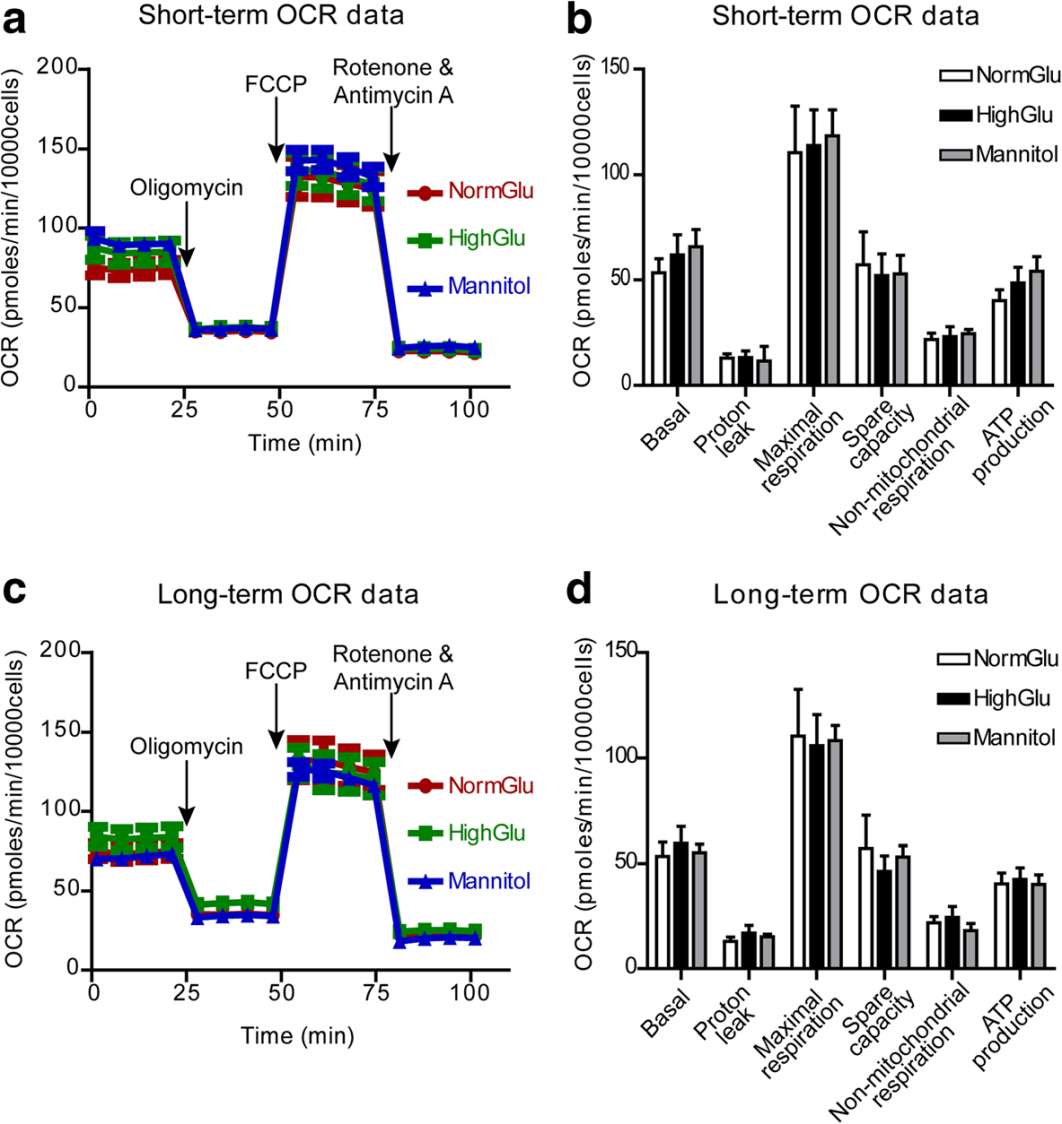
HighGlu treatments has no effect on the mitochondrial oxidative phosphorylation of BMDMs**.** Bone marrow cells were cultured for 7 days under NormGlu, HighGlu or Mannitol conditions (long-term treatments). For short-term HighGlu and Mannitol, cells differentiated under NormGlu, were exposed to HighGlu or Mannitol for 24 h, respectively. The Mito stress assay was performed using the seahorse XFe96 Analyzer. **a** Representative profile after Mito stress assay showing the OCR of BMDMs exposed to short-term NormGlu (red), HighGlu (green) or Mannitol (blue). **b** Graph showing basal OCR, proton leakage, maximal respiration, spare capacity, non-mitochondrial respiration and ATP production of BMDMs after acute NormGlu (white bars), HighGlu (black bars) and Mannitol (grey bars) treatments. **c** Representative profile after Mito stress assay showing the OCR of long-term treated BMDMs. **d** Graph showing the different aspects of mitochondrial oxidative phosphorylation pathway of BMDMs after long-term exposure to the different conditions. Data are represented as mean ± SEM. One-way ANOVA followed by Tukey’s multiple comparison post hoc test was performed

The Cell Mito Stress assay showed that the basal respiration rates were at similar levels in macrophages generated under the different conditions (Fig. 6a, c). Blocking ATP synthase using oligomycin allows the measurement of ATP production by mitochondria. Macrophages generated under different conditions had the same levels of mitochondrial ATP production (Fig. 6). In addition, the proton leakage, maximal respiration, spare capacity, non-mitochondrial respiration (Fig. 6b, d) were comparable in different macrophages.

Our results suggest that mitochondrial oxygen consumption was not affected by short-term or long-term high glucose treatment. The reduced glycolysis might contribute to high glucose-mediated altered macrophage functions.

## Discussion

In this study, we show that BMDMs generated under long-term high glucose conditions pose a number of intrinsic changes compared to cells generated under normal glucose conditions. Under naïve conditions BMDMs express higher levels of IL-1β and upon LPS + IFNγ stimulation they produce higher levels of TNFα but lower levels of nitric oxide. They also express higher levels of Arg-1 and IL-10 upon IL-4 stimulation compared with BMDMs generated under normal glucose conditions. The phagocytosis and bactericidal activity of these cells appear to be impaired. The phenotype and function of these long-term high glucose treated cells mirror the monocyte/macrophage functions observed in diabetic animals and patients, i.e. they are pro-inflammatory and contribute to the development of diabetic complications, but have reduced defence and tissue repair capacities. This in vitro system is therefore, a good model to study diabetes-mediated immune dysfunction.

Dysregulated innate immune activation is known to contribute to diabetic complications [1, 5, 6, 12]; whether this is due to the intrinsic change of innate immune cells or the noxious tissue microenvironment remains elusive. Results from diabetic animals and patients are controversial. The volume of bone marrow and the hematopoietic fraction were reduced in STZ-induced diabetic mice [13]. The populations of circulating F4/80^+^, CD11b^+^, CCR2^+^ or Ly6G^+^ cells in diabetic mice were reported to be reduced in some studies [14], but increased in others [6]. Mononuclear cells from diabetic patients secrete less IL-1 and IL-6 after LPS stimulation [15, 16]. Increased glycation is known to inhibit IL-10 and TNFα production by immune cells [17], which suggests that the lower cytokine production may be a consequence of diabetes-mediated intrinsic defect in these cells. In our study, long-term HighGlu did not affect the differentiation of bone marrow cells into F4/80^+^ macrophages, but the function of these cells appears to be altered (see below).

Nitric oxide (NO) is a signalling molecule critically involved in inflammation [18, 19]. Sustained production of NO endows macrophages with cytostatic or cytotoxic activity against viruses, bacteria and other external pathogens [20]. BMDMs cultured under long-term high glucose conditions produced less NO upon LPS + IFNγ stimulation, although the mRNA expression levels of iNOS did not show any significant reduction. It is possible that other isoforms of NOS (e.g., eNOS) or the availability of arginine in long-term high glucose treated macrophages may be affected. Additionally, the long-term high glucose may also affect the NO production at posttranscriptional, translational and posttranslational levels [21] . They also express lower levels of IL-6 and IL-12p40 and have impaired phagocytic and bactericidal functions. Reduced NO production has been reported in high glucose treated human macrophage J774 cells [22]. Increased NO production [23] and lower ROS production [24] has been observed in peritoneal macrophages from diabetic rats. BMDMs and peritoneal macrophages from the NOD mice had reduced ability to phagocytise apoptotic cells [25–27]. Reddy and colleagues [28] reported reduced expression of iNOS, IL-10 and Arg-1 in BMDM from db/db mice. All these results suggest that diabetes or long-term high glucose down-regulates iNOS expression and reduces NO production. The molecular mechanism underlying the differential responses to cytokine stimulations between short-term and long-term HighGlu treated macrophages remains elusive. We believe that the long-term HighGlu treated macrophages resemble their in vivo counterparts in diabetic conditions.

Despite reduced NO production following long-term HighGlu treatment, these cells express higher levels of TNFα and IL-1β, suggesting a pro-inflammatory phenotype. This agrees with a previous study, which showed that the myeloid cells recruited to wounds of diabetic mice are intrinsically primed to be more pro-inflammatory than those of non-diabetic mice [29]. TNFα and IL-1β were also higher in cultures of peritoneal macrophages in STZ-induced diabetic rats comparing to control rats [23]. High glucose leads to increased production of IL-1β and TNFα via the NF-κB pathway [30, 31]. Interestingly, the long-term HighGlu treated macrophages also express higher levels of Arg-1 and IL-10 following IL-4 stimulation, suggesting an enhanced M2 phenotype. A previous study by Cucak and colleagues [32] reported an M2-type phenotype in macrophages from 28-week db/db mice. Our data suggest that long-term high glucose sensitizes macrophages to cytokine stimulations i.e., they present exaggerated responses to both Th1- and Th2-type cytokine stimulation, which may contribute to the dysregulated immune response observed in diabetic patients.

The molecular mechanism underlying long-term HighGlu-mediated alteration of macrophage function remains unknown. Glucose uptake in macrophages is mediated predominately by glucose transporter 1 [33], which does not rely on insulin signalling. We have found that the HighGlu treatment impairs macrophage glycolytic capacity and glycolytic reserve without affecting mitochondrial ATP production and oxidative respiration. Glycolytic capacity is a measure of the maximum rate of conversion of glucose to pyruvate or lactate by a cell, and the difference between basal glycolysis and glycolytic capacity is known as glycolytic reserve. The basal glycolysis did not differ between BMDMs under NormGlu and HighGlu. Therefore, the reduction in glycolytic reserve may contribute predominately to impaired glycolytic capacity. Glycolytic reserve is an important bioenergy source in response to increases in ATP demand. Our results suggest that long-term HighGlu treatment may limit macrophage energy supply when additional ATP is needed. The LPS-induced proinflammatory macrophages are known to be fuelled by glycolysis [34]. High levels of glycolysis generate NADPH to support NO and ROS production. The reduced glycolytic capacity may explain the lower levels of NO production and impaired bactericidal activity in long-term HighGlu treated macrophages. It may also be related to the reduced phagocytosis of these cells, as we have recently shown that macrophage phagocytic function is directly associated with glycolytic function [35].

## Conclusions

Our study shows that long-term HighGlu sensitizes macrophages to cytokine stimulations, but reduces phagocytosis and bactericidal function of macrophages. The latter may be related to the reduced glycolytic capacity and glycolytic reserve of these cells. Further understanding of the link between altered glucose metabolism and dysregulated macrophage function in diabetes will be important to uncover the immunopathogenesis of diabetic complications.

## Methods

### Animals

C57BL/6 J mice were purchased from Harlan and housed under specific pathogen-free conditions with free access to normal chow and water. Diabetes was induced in 12-week male C57BL/6 J mice by intraperitoneal injection of streptozotocin (STZ) at 50 mg/kg for 5 consecutive days [36]. Non-fasting glucose level was measured 1 week after the last injection; mice with glucose level over 13 mmol/l were considered diabetic. If the glucose level was reversed back to normal within 2 months after STZ injection, the mice were considered as ex-diabetic. Body weight was monitored monthly (Additional file 2: Figure S2). Mice were sacrificed 3 months after STZ injection and peritoneal macrophages were harvested from diabetic mice (*n* = 3), age-matched controls (n = 3) and ex-diabetic mice (n = 3). Mice were euthanized by exposure to CO_2_. All animal procedures were approved by the UK Home Office under the Home Office Animal Act (1986) (PPL 2773).

### Bone marrow-derived macrophage culture

Bone marrow derived macrophages (BMDMs) were cultured from 8 to 12-week-old C57BL/6 J mice using a protocol described previously [37]. Briefly, bone marrow cells were cultured in complete DMEM (Thermo Fisher Scientific, Waltham, Massachusetts, USA) supplemented with 10% heat-inactivated foetal bovine serum (FBS) and 20% L929-conditioned media as a source of macrophage colony-stimulating factor (M-CSF) [37]. The phenotype of BMDMs was confirmed by flow cytometry as being F4/80^+^ and CD11b^+^ (data not shown).

### High glucose treatment of BMDMs

**Fig. 7:**
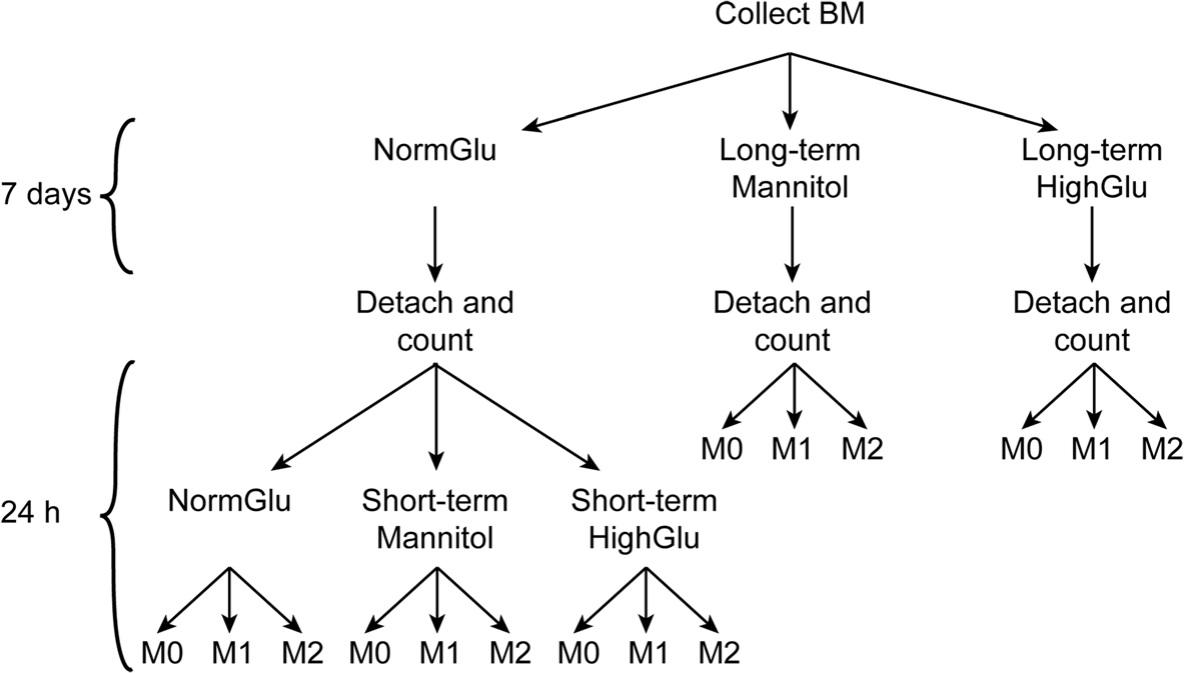
Schematic illustration showing the experimental setup. BM was collected and cultured under NormGlu, HighGlu or Mannitol conditions for 7 days (long-term treatments). BMDMs were then counted and stimulated with LPS + IFNγ (M1) or IL-4 (M2) for 24 h. BMDMs differentiated under NormGlu were exposed to short-term HighGlu or Mannitol, along with M1 or M2 stimulations for 24 h

Three types of culture media were used in the in vitro studies: normal glucose DMEM (NormGlu), high glucose DMEM (HighGlu), osmolarity control (Mannitol) medium. NormGlu is the standard DMEM (5.5 mM glucose, Thermo Fisher Scientific). HighGlu is the standard DMEM supplemented with additional 25 mM glucose. Previous studies have used glucose concentration ranging from 25 to 40 mM to mimic diabetic condition in cell cultures [38, 39]. Osmolarity control medium (Mannitol) is the standard DMEM supplemented with 25 mM Mannitol [40]. BMDMs were treated for short-term (24 h) or long-term (7 days). Figure 7 shows the experimental design and work flow.

#### Short-term treatment

BMDMs were generated from bone marrow cells under NormGlu condition. The cells were collected on day 7 and plated in 12- or 96-well plates with NormGlu, HighGlu or Mannitol for further investigations.

#### Long-term treatment

BMDMs were generated under NormGlu, HighGlu or Mannitol conditions. On day 7, cells were harvested and plated in 12- or 96-well plates for further investigations.

### Macrophage polarization with cytokines

BMDMs were seeded in 12-well plates at a density of 5 × 10^5^ cells per well. The cells were polarized into M1 with 50 ng/ml LPS (Sigma-Aldrich, St. Louise, Missouri, USA) + 100 ng/ml IFNγ (Bio-Techne, Minneapolis, Minnesota, USA) or M2 with 20 ng/ml IL-4. (Bio-Techne, Minneapolis, Minnesota, USA) The supernatants were collected 24 h after cytokine stimulation and nitric oxide (NO) was measured using the Griess Reagent System (Promega, Madison, Wisconsin, USA), according to manufacturer’s instructions. IL-1β in BMDM supernatants was measured using the mouse IL-1 beta/IL-1F2 DuoSet ELISA kit (DY401, Bio-Techne, Minneapolis, Minnesota, USA) according to manufacturer’s instructions. Cells were collected for flow cytometry, protein or RNA extraction.

### Assessment of BMDMs cell death and proliferation

Cell death of BMDMs cultured under different glucose conditions (NormGlu, HighGlu or Mannitol control) were measured using Thiazolyl Blue Tetrazolium Blue (MTT, Sigma-Aldrich, St. Louise, Missouri, USA) [41] and TUNEL assay (In Situ Cell Death Detection Kit, TMR red, Roche, Basel, Switzerland), according to manufacturer’s instructions. Cell proliferation was assessed by Ki67 (1:100, clone: SolA15, Thermo Fisher Scientific) immunofluorescence, using a standard protocol [42]. For these assays, cells were counted on the day of bone marrow extraction and 1 × 10^4^ cells were plated in a 96-well plate. After 7 days of differentiation the assays were performed.

For MTT assay, cells were incubated in 0.5 mg/ml MTT for 4 h at 37 °C. The MTT solution was then replaced by 200 μl DMSO per well and cells were incubated for 30 min at 37 °C until the purple formazan crystals were dissolved. Absorbance was measured at 562 nm using the Fluostar Omega plate reader (BMG Labtech, Ortenberg, Germany). For the TUNEL assay, cells were fixed in 2% PFA for 15 min at room temperature. After several washes with PBS, cells were incubated with the TUNEL-mix for 1 h at 37 °C. After washes with PBS, cells were imaged using an IX51 inverted fluorescent microscope (Olympus, Southend, UK). Cells were treated with DNase (Thermo Fisher Scientific) for 10 min at room temperature, before the addition of the TUNEL-mix, as a positive control.

### Western blot

Total protein was extracted in RIPA buffer supplemented with 10% proteinase inhibitor cocktail (Sigma-Aldrich) and quantified using the BCA assay (Thermo Fisher Scientific). The same amount of denatured proteins from each treatment group was separated on a 12% SDS-PAGE gel and transferred onto polyvinylidene fluoride (PVDF) membranes. Membranes were incubated with antibody against mouse Caspase-3 (1:100, Millipore, Darmstadt, Germany) at 4 °C overnight, followed by anti-rabbit-HRP (1:5000, Agilent Technologies, Santa Clara, California, USA) for 1 h at room temperature. Protein from BMDMs stimulated with 20 ng/ml TNFα for 3 h at 37 °C was used as a positive control for the cleaved Caspase-3. Membranes were developed using enhanced chemiluminescence (Clarity™ Western ECL Blotting Substrates, BioRad, Hercules, California, USA) and corresponding bands were detected using Syngene G-Box imaging system (Syngene, Cambridge, UK).

### Quantitative real time RT-PCR

Total RNA was extracted using the RNeasy mini kit (Qiagen, Hilden, Germany) and cDNA was synthesised using Superscript II Reserve Transcriptase (Thermo Fisher Scientific) with random primers (Thermo Fisher Scientific) following the manufacturer’s instructions. QRT-PCR was performed using the LightCycler 480 SYBR Green Master Mix (Roche). The primer sequences used in the study are shown in Table 1. Relative gene expression was normalised to 18S ribosomal RNA (Rn18s) using the 2^-ΔΔCT^ method.

**Table 1:** Primers used for RT-PCR

Name	Forward	Reverse
Arg-1	TTATCGGAGCGCCTTTCTCAA	TGGTCTCTCACGTCATACTCTGT
IFNγR1	GGTTGCTCCTCTTACCGTCT	GTGCGGTGTGACAAGTGAAT
IFNγR2	TGCTTCACCCTGTTCCTCAA	CTGGTTCACGGTGTTTGGAG
IL-10	TGCAGGACTTTAAGGGTTACTTGG	GGCCTTGTAGACACCTTGGTC
IL-12p40	GACATCATCAAACCAGACCCGCC	GCCTTTGCATTGGACTTCGGT
IL-1β	TCCTTGTGCAAGTGTCTGAAGC	ATGAGTGATACTGCCTGCCTGA
IL4ra	TGGCTGCTGACCTGGAATAA	TCAGCCTGGGTTCCTTGTAG
IL-6	TCTGCAAGAGACTTCCATCCAGT	TCTGCAAGTGCATCATCGTTGT
iNOS	GGCAAACCCAAGGTCTACGTT	TCGCTCAAGTTCAGCTTGGT
Rn18s	AGGGGAGAGCGGGTAAGAGA	GGACAGGACTAGGCGGAACA
TLR-4	TCCTGGCTAGGACTCTGATCAT	TCCAGCCACTGAAGTTCTGA
TNFα	GCCTCTTCTCATTCCTGCTT	CTCCTCCACTTGGTGGTTTG

### Flow cytometric analysis

Single-cell suspensions were stained for cell surface markers using specific antibodies following protocols published previously [43]. All samples were analysed using a FACS Canto II (BD biosciences, UK) and data were processed using FlowJo Software (version 7 for Windows, Tree Star, Ashland, OR, USA). The following antibodies were used: F4/80 (clone: BM8, Thermo Fisher Scientific), CD16/32 (clone: 2.4G2, Thermo Fisher Scientific); CD36 (clone: HM36, Biolegend, San Diego, California, USA), CD206 (clone: C068C2, Biolegend).

### Macrophage phagocytosis

Phagocytosis was conducted using the pHrodo *S. aureus* bioparticles conjugated phagocytosis kit (Thermo Fisher Scientific) following the manufacturer’s instructions [35]. Briefly, BMDMs or peritoneal macrophages were seeded onto 96-well black-walled plates at a density of 5 × 10^4^ per well for 24 h before adding the bioparticles. Culture medium with bioparticles only was served as a negative control. Fluorescence intensity was measured 1 h, 1.5 h and 2 h after the addition of bioparticles using the Fluostar Omega plate reader (BMG Labtech). The net fluorescence intensity was the difference between the fluorescence intensity from the experimental wells and that from the negative control wells [35].

### Bacterial killing assays

A colony of *Pseudomonas aeruginosa* PAO1 was selected from a cetrimide agar plate and inoculated into 10 mL of LB broth (Sigma-Aldrich). This was grown overnight at 37 °C in an orbital shaker at 160 rpm. The cells were harvested by centrifugation in an Eppendorf centrifuge 5810R at 4000 rpm for 10 mins and resuspended in 40 ml of fresh LB broth. They were grown to log phase for 4–6 h at 37 °C on a rotary shaker at 160 rpm. The optical density of the bacterial culture was measured at 600 nm (WPA biowave).

To assess their bactericidal activity, 1 × 10^6^ BMDMs were plated in 12-well plates. *P. aeruginosa* was added to the wells at multiplicity of infection (MOI) 1:10 (1 × 10^7^ CFU/ml). The BMDM/*P. aeruginosa* co-culture was incubated for 1 h at 37 °C. The coculture was then centrifuged at 1500 rpm for 5 min and the supernatant removed. The supernatant was serial diluted in PBS at 10^− 4^, 10^− 5^, 10^− 6^ and 10^− 7^ and plated onto cetrimide agar (Sigma-Aldrich). Saponin (Sigma-Aldrich) at 1:1000 dilution in PBS was added to all pellets and incubated for 5 min. The digested pellets were serial diluted in PBS at 10^− 4^, 10^− 5^, 10^− 6^ and 10^− 7^ and plated onto cetrimide agar. All cetrimide plates were incubated for 18 h and CFU/mL were then calculated.

### Bioenergetic profile of BMDMs

BMDMs metabolic profiles were assessed using Seahorse XF Glycolysis Stress Test kit and Cell Mito Stress Test kit with a Seahorse XFe96 Analyzer (Agilent Technologies) following manufacturer’s instructions. Glycolytic activities were assessed by measuring the Extracellular Acidification Rates (ECAR) and mitochondrial activities were assessed by measuring the Oxygen Consumption Rates (OCR). Data were normalised with the total cell number and expressed as mpH/min/10^4^ cells for ECAR or pmol/min/10^4^ cell for OCR.

### Statistical analysis

Data were presented as mean ± standard error (SEM). Unpaired, two-tailed Student’s t test was used for two-group comparisons. One-way ANOVA was used to compare the means of multiple groups; Tukey’s multiple comparisons were used for post hoc test when samples had equal variances. Student t test with Welch’s correction was employed when the equal variance assumption was not met. *P* < 0.05 was considered statistically significant.

## Additional files

Additional file 1: **Figure S1**. Phagocytosis of BMDMs exposed to short-term HighGlu. Bone marrow cells were differentiated under NormGlu and then exposed to HighGlu for 24 h. Phagocytosis was assessed using pHrodo *S. aureus* bioparticles. Data are represented as mean ± SEM. Two-way ANOVA with bonferroni correction was performed. (TIF 257 kb)

Additional file 2: **Figure S2**. Body weights of control and diabetic (DM) mice, before and after STZ injections. Mice were weighted (time point 0) and STZ (50 mg/kg) was injected for 5 consecutive days. One week after the last STZ injection (time point 1), mice had elevated blood glucose levels (diabetic-DM). Weights were monitored every 4 weeks until the end of the experiment. Data are represented as mean ± SEM. Two-way ANOVA with bonferroni correction was performed; *** *p* < 0.001. (TIF 256 kb)

## Abbreviations

ATP: Adenosine triphosphate
BMDMs: Bone marrow derived macrophages
ECAR: Extracellular acidification rate
HighGlu: High glucose (30.5 mM)
NormGlu: Normal glucose (5.5 mM)
OCR: Oxygen consumption rate
ROS: Reactive oxygen species
STZ: Streptozotocin
