# Supplementary figures and images for: Sustained high glucose exposure sensitizes macrophage responses to cytokine stimuli but reduces their phagocytic activity
Source: BMC Immunol. 2018 Jul 11;19:24. doi: 10.1186/s12865-018-0261-0 (PMC6042333; doi:10.1186/s12865-018-0261-0)

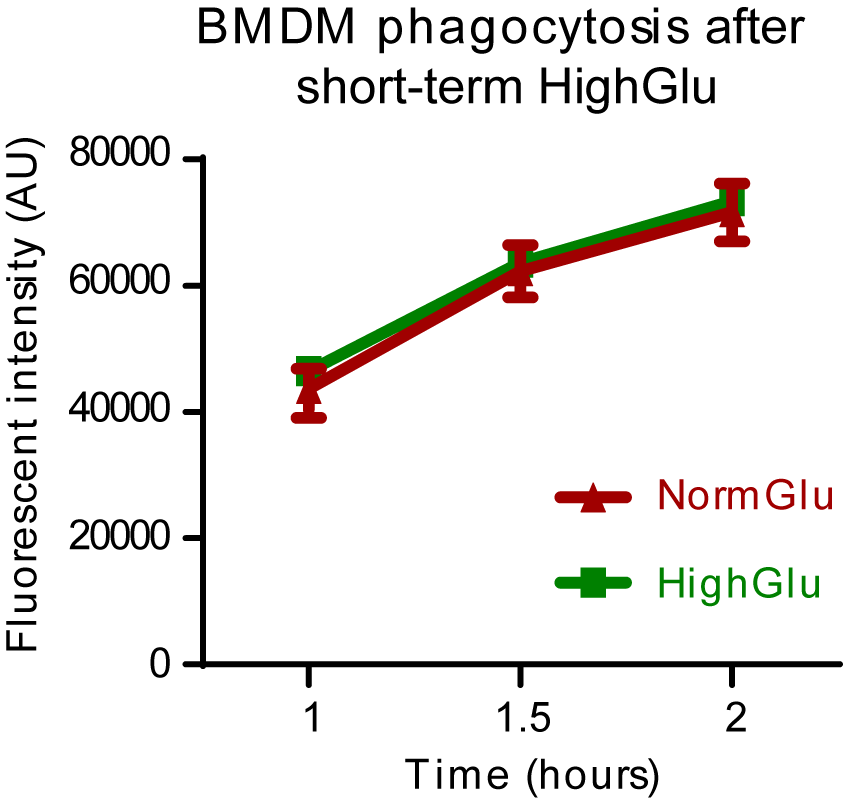

Supplement: Supplementary file 1 — Figure S1. Phagocytosis of BMDMs exposed to short-term HighGlu. Bone marrow cells were differentiated under NormGlu and then exposed to HighGlu for 24 h. Phagocytosis was assessed using pHrodo S. aureus bioparticles. Data are represented as mean ± SEM. Two-way ANOVA with bonferroni correction was performed. (TIF 257 kb) [file 12865_2018_261_MOESM1_ESM.tif]

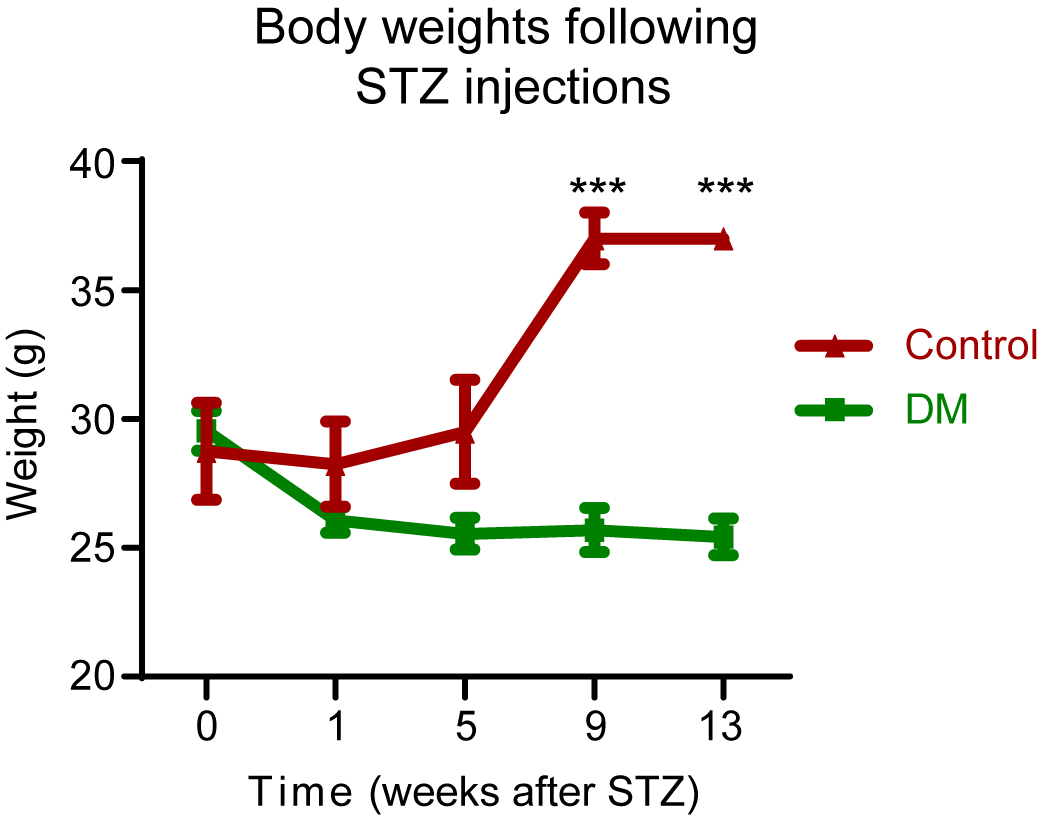

Supplement: Supplementary file 2 — Figure S2. Body weights of control and diabetic (DM) mice, before and after STZ injections. Mice were weighted (time point 0) and STZ (50 mg/kg) was injected for 5 consecutive days. One week after the last STZ injection (time point 1), mice had elevated blood glucose levels (diabetic-DM). Weights were monitored every 4 weeks until the end of the experiment. Data are represented as mean ± SEM. Two-way ANOVA with bonferroni correction was performed; *** p < 0.001. (TIF 256 kb) [file 12865_2018_261_MOESM2_ESM.tif]
